# A Critical Role of a Cellular Membrane Traffic Protein in Poliovirus RNA Replication

**DOI:** 10.1371/journal.ppat.1000216

**Published:** 2008-11-21

**Authors:** George A. Belov, Qian Feng, Krisztina Nikovics, Catherine L. Jackson, Ellie Ehrenfeld

**Affiliations:** 1 National Institute of Allergy and Infectious Diseases, National Institutes of Health, Bethesda, Maryland, United States of America; 2 Laboratoire d'Enzymologie et Biochimie Structurales, CNRS, Gif-sur-Yvette, France; University of California San Francisco, United States of America

## Abstract

Replication of many RNA viruses is accompanied by extensive remodeling of intracellular membranes. In poliovirus-infected cells, ER and Golgi stacks disappear, while new clusters of vesicle-like structures form sites for viral RNA synthesis. Virus replication is inhibited by brefeldin A (BFA), implicating some components(s) of the cellular secretory pathway in virus growth. Formation of characteristic vesicles induced by expression of viral proteins was not inhibited by BFA, but they were functionally deficient. GBF1, a guanine nucleotide exchange factor for the small cellular GTPases, Arf, is responsible for the sensitivity of virus infection to BFA, and is required for virus replication. Knockdown of GBF1 expression inhibited virus replication, which was rescued by catalytically active protein with an intact N-terminal sequence. We identified a mutation in GBF1 that allows growth of poliovirus in the presence of BFA. Interaction between GBF1 and viral protein 3A determined the outcome of infection in the presence of BFA.

## Author Summary

All positive strand RNA viruses replicate their genomes in association with membranous structures that are formed after infection by remodeling pre-existing cellular organelles. The role of membranes and the mechanisms exploited by viral proteins to orchestrate the formation and functioning of viral membranous replication complexes are largely unknown. Poliovirus replication is severely suppressed by brefeldin A (BFA), a well-known inhibitor of the cellular secretory pathway. Three cellular proteins (GBF1, BIG1 and BIG2) that activate small GTPases called Arfs, whose activity is necessary for normal functioning of the secretory pathway, are known targets of BFA. Here we demonstrate that poliovirus utilizes the GBF1-dependent Arf activation pathway for its replication. Our data explain the mechanism of BFA inhibition of poliovirus replication by demonstrating that viral protein 3A binds and recruits GBF1 to membranes that support viral RNA synthesis. Inactivation of GBF1 by BFA prevents Arf activation and recruitment, and prevents formation of functional replication complexes. Surprisingly, formation of membranous structures morphologically similar to viral replication complexes occurs in the presence of BFA, although these structures do not function in the synthesis of viral RNA. Other plus strand RNA viruses are known to exhibit sensitivity to BFA and our data suggest that hijacking of the Arf activation pathway may be a common feature shared by diverse groups of viruses.

## Introduction

All known positive strand RNA viruses replicate their genomes in association with remodeled cellular membranes. Assembly of replication complexes on membranes is believed to have several advantages. Membranes provide a scaffold that increases the local concentration of proteins involved in replication and facilitates the proper topological orientation of replication complex components. The association with membranes protects replicating RNA from cellular nucleases, and may also prevent induction of cellular innate immune responses by confining dsRNA or other signaling intermediates [1].

**Figure 1:**
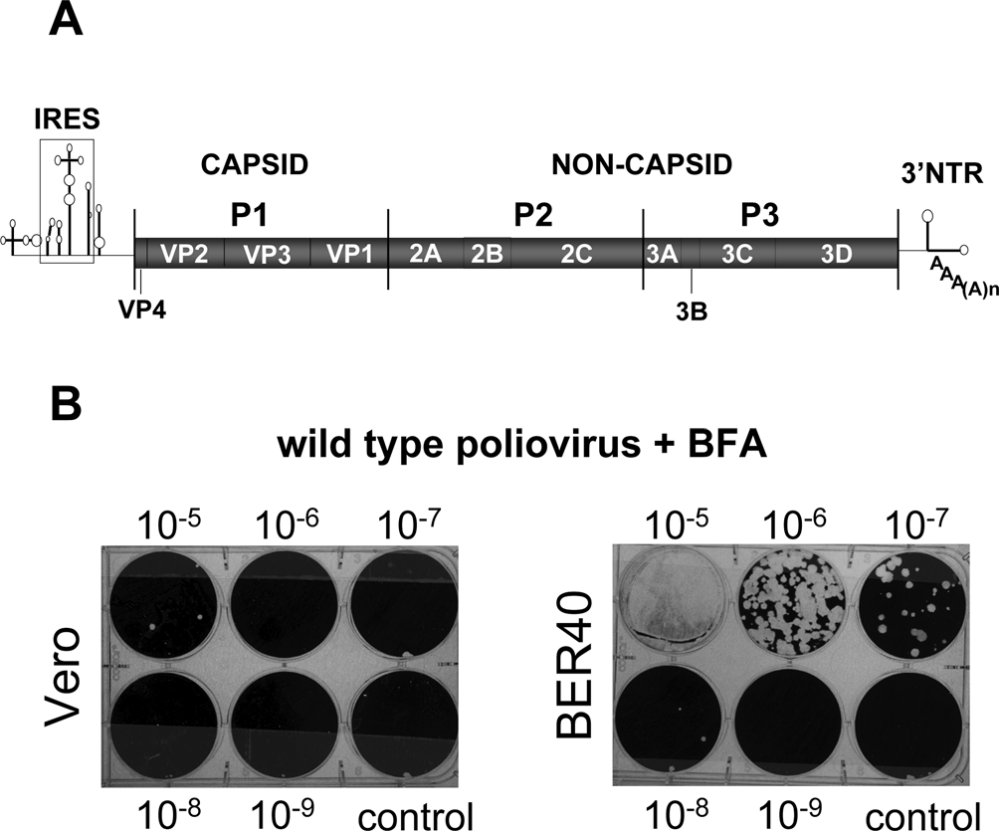
Inhibition of poliovirus growth by BFA depends on the host cell. A. Schematic map of the poliovirus genome. B. Poliovirus was propagated on Vero or BER-40 cells for 6 h in the presence of 2 µg/ml BFA. Virus yields were measured by plaque assay on HeLa cell monolayers. Dilutions of the infected cell suspensions are indicated.

Poliovirus is a member of the *Picornaviridae* family, which consists of small, non-enveloped positive strand RNA viruses that include numerous human and veterinary pathogens, such as polio, rhinovirus (common cold virus), hepatitis A virus, and foot and mouth disease virus. The poliovirus genome is a single RNA molecule of about 7500 nt in length which is directly translated in an infected cell into a single polyprotein that undergoes immediate processing *in cis* and *in trans* by three virus-encoded proteases into a cascade of intermediates and mature polypeptides. Non-structural proteins, necessary for RNA replication, are encoded in the P2-P3 region of the genome, while coding sequences for structural proteins, necessary for packaging of progeny RNA but dispensable for replication, are located in the P1 region (Fig. 1A). Infection of cells with poliovirus results in rapid and massive reorganization of virtually all intracellular membranes except for mitochondria, into clusters of tightly-associated vesicles of heterogeneous size which harbor viral replication complexes on their surfaces [2],[3],[4]. These replication complexes have been shown to be associated with all of the non-structural viral proteins from the P2 and P3 coding region [5],[6]. Such massive rearrangements in cellular membrane organization likely require major rewiring of normal membrane metabolism, but the molecular mechanisms underlying induction, formation and functioning of poliovirus membranous replication complexes remain largely unknown. It has been shown that at the early stages of poliovirus infection non-structural virus protein 2B co-localizes with COPII-coated vesicles, budding from ER exit sites [7]. These data together with the observations that poliovirus-induced vesicles are often found in electron micrographs close to the remnants of ER [8] suggest that the COPII-dependent mechanism of vesicle formation may contribute to the development of viral replication complexes. However, Shlegel et al. have identified markers not only from the ER, but also from Golgi and lysosomes, present on polio-induced vesicles. It was proposed that autophagy-like processes may be involved in membrane remodeling in polio infected cells, which would also explain the large proportion of double membrane vesicles observed by electron microscopy of infected cells [9].

Another important clue towards possible cellular pathways involved in formation of polio replication complexes comes from the sensitivity of poliovirus infection to brefeldin A (BFA) [10],[11]. Interestingly, neither formation of COPII-coated vesicles nor autophagy are sensitive to BFA [12],[13],[14], suggesting that these two processes do not fully account for all cellular pathways exploited by poliovirus for its replication. BFA is a fungal metabolite, specifically targeting the activation of small cellular GTPases, Arfs, which are key regulators of the cellular secretory pathway. The inactive, cytoplasmic GDP-bound form of Arf, upon nucleotide exchange to GTP, undergoes conformational changes that allow Arf-GTP to bind membranes. Arf-GTP is referred to as “activated”; it initiates formation of secretory vesicles and regulates cytoskeleton functions and lipid metabolism [15]. Conversion of Arf-GDP into Arf-GTP requires the activity of guanine nucleotide exchange factors (GEFs). In human cells BFA inhibits the function of three GEFs – GBF1, BIG1 and BIG2 – by stabilizing transient complexes formed between the GEF and Arf-GDP. The specificity of BFA action on these GEFs is provided by the sequence of their Sec7 domains, directly involved in Arf activation [16].

We have shown previously that Arf-GTP accumulates on membranes in poliovirus- infected cells. Surprisingly, in an *in vitro* reaction, expression of two distinct viral proteins – 3A, a small membrane-binding protein, and 3CD, a soluble protein with protease activity – was sufficient to induce Arf translocation to membranes. We demonstrated that these two proteins specifically engage different cellular GEFs: 3A induces translocation of GBF1 to membranes while 3CD results in association with membranes of BIG1 and BIG2 [17],[18],[19]. Thus, all three mammalian BFA-sensitive GEFs could be involved in poliovirus replication. Wessels et al. have demonstrated that direct interaction between GBF1 and 3A from poliovirus or coxsackie virus B3, a close relative of poliovirus, is responsible for association of GBF1 with membranes in 3A-expressing cells. When 3A was expressed individually, binding of 3A to GBF1 resulted in inhibition of the Arf-activating function of GBF1 [20],[21]. This mechanism was proposed to explain an established phenomenon of inhibition of cellular protein secretion in polio- and coxsackievirus-infected cells, which had been shown previously to be caused by 3A expression [22],[23],[24]. In this paper we show that in the context of normal virus replication, functional GBF1 is required for successful virus propagation, and GBF1-3A interactions determine the outcome of infection in the presence of BFA. Surprisingly, the BFA-sensitive step in poliovirus replication was not the morphological remodeling of cellular membranes, but the functioning of replication complexes, suggesting strong dependence of poliovirus RNA replication on components of the host membrane traffic machinery.

## Results

### Sensitivity of poliovirus to BFA depends on the host cell

To determine whether host cell factors contribute to BFA sensitivity of poliovirus replication, we took advantage of an available BFA-resistant cell line, BER-40, which was isolated from parental Vero cells after mutagenesis and subsequent passaging in the presence of BFA [25]. Vero cells are routinely used in large-scale viral vaccine production, and they support robust replication of poliovirus. Figure 1B shows that while replication of poliovirus in Vero cells was severely inhibited by BFA, the BFA-resistant derivative of Vero cells, BER-40, was able to support replication of the virus in the presence of the inhibitor. These results demonstrate that some cellular BFA-sensitive process is required for successful propagation of poliovirus in Vero cells.

### Mutation in GBF1 is responsible for BFA resistance of BER-40 cells

**Figure 2:**
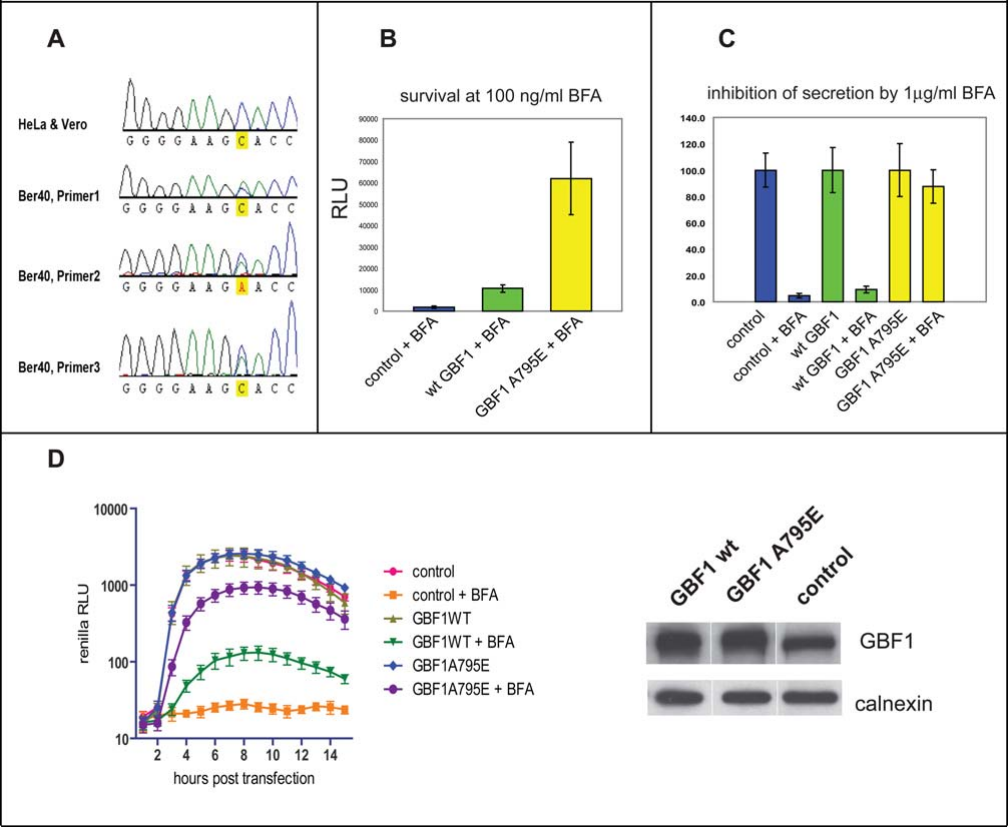
GBF1 sequence determines BFA resistance of cells and virus. A. Sequencing chromatograms of GBF1 Sec7 domain from Vero and BER-40 cells. Results from analyses with three different primers are shown. B. GBF1 A795E mutant confers BFA resistance to HeLa cells. Cells were transfected with either a control vector, vector expressing wild type YFP-GBF1 fusion or vector expressing YFP-GBF1 A795E mutant fusion, and subsequent cell growth in the presence of BFA was measured by a luminescent cell viability assay. C. Expression of GBF1 A795E mutant rescues protein secretion in the presence of BFA. Cells were co-transfected with pCMV-Gluc vector expressing secreted *Gaussia* luciferase and with either control plasmid or with vectors expressing wild type GBF1-YFP or GBF1 A795E-YFP fusions. The amount of secreted protein observed in each sample without BFA was defined as 100%. D. GBF1 A795E mutant rescues replication of poliovirus in the presence of BFA. A polio *Renilla* luciferase replicon was introduced in HeLa cells previously transfected with vectors expressing wt YFP-GBF1 fusion, YFP-GBF1 A795E mutant or with an empty vector. BFA was added where indicated at 1 µg/ml concentration at the time of replicon transfection, and polio RNA replication was measured by luciferase assay. Expression of the GBF1 proteins was measured by Western blot; calnexin staining was used as a loading control (panel D, right).

To identify the BFA-sensitive host factor involved in poliovirus replication, we decided to investigate the mechanism of resistance of BER40 cells to the inhibitor. Previous attempts to identify the determinants of BFA resistance in BER40 cells were unsuccessful [25],[26],[27]. We examined the gene sequences coding for all three high molecular weight Arf GEFs – GBF1, BIG1 and BIG2 – that are known to be targets of BFA, to determine whether mutations were present in BER-40 cells compared with the parental Vero cell line. Formation of a stable complex between BFA, a sensitive ArfGEF and Arf-GDP is determined by specific amino acids in the Sec7 domains of the ArfGEFs; the rest of the protein does not participate in this interaction [28]. We amplified the Sec7 domain coding sequences of BIG1, BIG2 and GBF1 from mRNA isolated from Vero and BER-40 cells. Sequence analysis showed that while BIG1 and BIG2 Sec7 domains were identical in both cell lines, BER-40 cells contained two species of GBF1 Sec7 sequences (Fig. 2A). One corresponded to the same sequence found in the parental Vero cells, while the other coded for a substitution of the A in GBF1 residue 795 to E (A795E). The two gene sequences likely arise from genetic heterozygosity in the BER-40 cells rather than a mixture of two cell populations, since a sensitive population would be rapidly selected against in the presence of BFA. To confirm that this mutation was responsible for the BFA-resistant phenotype of BER40, we introduced it into an expression plasmid coding for YFP-GBF1 fusion protein, shown previously to be indistinguishable from the wild type GBF1 in intracellular localization and functional activities [29]. The mutated plasmid was used to transfect HeLa cells, a human cell line commonly used as a laboratory host for poliovirus and known to be highly sensitive to BFA. Transfection with the mutated plasmid conferred a greatly increased resistance to BFA, compared with cells transfected with an empty vector or cells transfected with a plasmid encoding the wild type GBF1 sequence (Fig. 2B). In addition to increased cell survival in the presence of BFA, the BFA-resistant form of GBF1 also rescued the functional properties of the cells' secretory pathway, which are known to be sensitive to BFA treatment [30]. HeLa cells were co-transfected with pGLUC plasmid, expressing *Gaussia* luciferase with a natural secretion signal, and with plasmids expressing wild type GBF1, A795E BFA resistant mutant GBF1, or an empty vector. As seen in Fig. 2C, expression of the A795E GBF1 mutant almost completely restored secretion in the presence of BFA. Thus, the mutation in the GBF1 Sec7 domain is the major determinant of BFA resistance in the BER-40 cell line and can transfer BFA resistance to HeLa cells.

### BFA-resistant, catalytically-active GBF1 rescues replication of poliovirus

**Figure 3:**
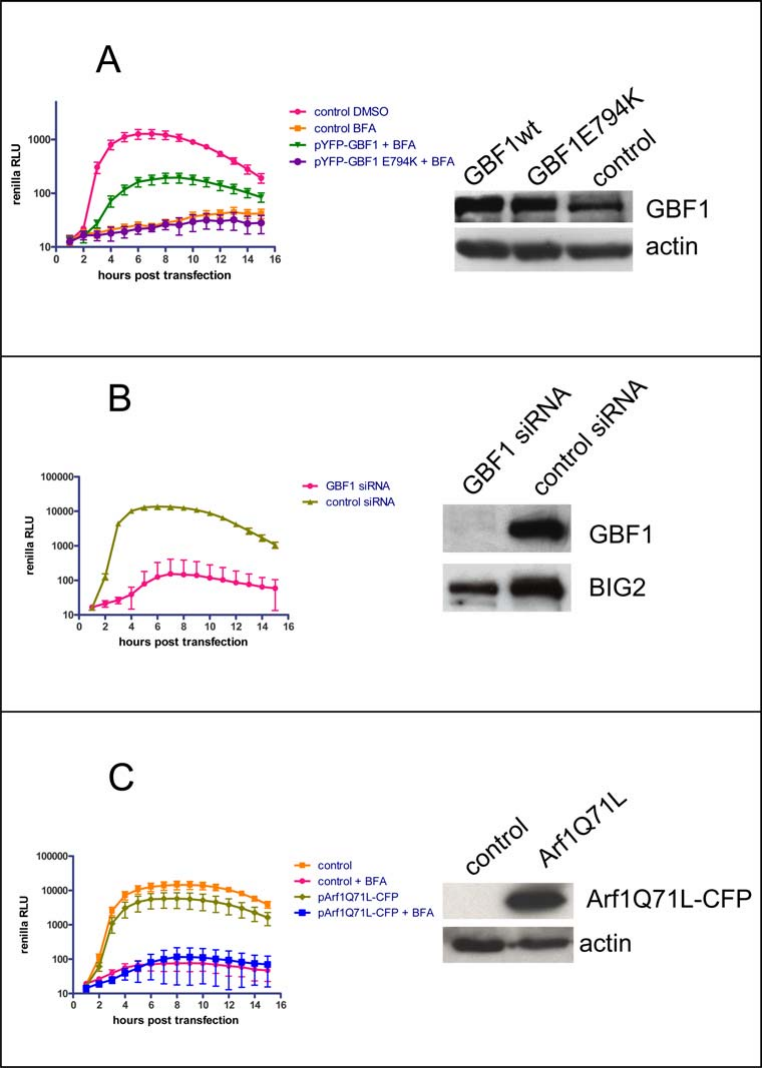
Requirement of active GBF1 for poliovirus replication in the presence of BFA. A. HeLa cells were transfected with a plasmid expressing active YFP-GBF1 fusion, inactive YFP-GBF1 E794K fusion or control vector one day prior to transfection with a polio replicon containing the *Renilla* luciferase gene, and incubated in the presence of 1 µg/ml of BFA where indicated. Luciferase activity was assayed as a measure of polio RNA replication. The Western blot on the right shows expression of GBF1 derivatives. Actin staining served as a loading control. B. HeLa cells were treated with either GBF1 or control non-specific siRNA for 3 days prior to transfection with the polio Renilla replicon. The Western blot shows the level of knock down of GBF1 protein. The same membrane was stripped and probed again with anti-BIG2 antibodies as a loading control. C. HeLa cells were transfected with either a control vector or plasmid pArf1 Q71L-CFP, expressing a constitutively activated form of Arf1-CFP fusion. The next day the cells were transfected with a polio replicon containing the *Renilla* luciferase gene, and incubated in the presence of 2 µg/ml of BFA where indicated. Luciferase activity was assayed as a measure of polio RNA replication. The Western blot on the right shows expression of Arf1 Q71L-CFP fusion. Actin staining serves as a loading control.

The GBF1 mutation in Ber40 cells conferred resistance of cell growth to BFA, and we predicted that this same mutation also was responsible for poliovirus growth in Ber-40 cells (see Fig. 1B). We have previously shown that ectopic expression of GBF1 can partially rescue poliovirus replication in the presence of BFA in HeLa cells [31]. To determine whether the growth of poliovirus in BER-40 cells in the presence of BFA was due to the mutated GBF1, we compared the replication of a poliovirus replicon in HeLa cells transfected with plasmids coding for either the wild type or the A795E form of GBF1. Figure 2D shows that replicon replication in the presence of BFA occurred with much greater efficiency in cells expressing GBF1 A795E. Equal amounts of wild type and mutant GBF1 proteins were synthesized, as measured by western blot analysis (Fig. 2D, right panel). Thus, the substitution of A795E found in the GBF1 sequence from BER-40 cells is responsible for resistance of both cell secretion and viability and poliovirus replication to BFA. We also tested whether the rescue of polio replication from BFA inhibition in HeLa cells was dependent on GBF1's ability to functionally activate Arf. To this end, we exploited another GBF1 mutation that encoded a protein with a single amino acid substitution in the Sec7 domain (E794K), which was shown previously to be inactive in Arf activation [32]. HeLa cells were transfected with expression plasmids for wild type GBF1 and for the E794K GBF1 mutant. While a polio replicon was able to replicate in the presence of BFA in cells expressing active GBF1, cells expressing the inactive GBF1 mutant were unable to support polio replication in the presence of the same concentration of inhibitor (Fig. 3A), demonstrating that the ability of GBF1 to activate Arf is required for polio replication.

### Depletion of GBF1 reduces polio replication

As a second approach to evaluate the requirement of GBF1 for poliovirus replication even in cells not treated with BFA, we depleted GBF1 levels in untreated HeLa cells with siRNA. The depletion of GBF1 was very effective after three days of siRNA treatment, and replication of the polio replicon was severely inhibited compared with cells treated with control siRNA (Fig. 3C). Upon completion of the replication experiments the cells treated with GBF1 siRNA showed some signs of cytotoxicity and lysis [33] which resulted in reduction in the amount of BIG2, another high molecular GEF, as well as other bands (not shown) on blots of lysates obtained from GBF1 siRNA-treated cells (Fig. 3B). However, expression levels of control proteins from plasmids introduced at the same time as the polio replicon were identical in control cells and cells treated with GBF1 siRNA (not shown), confirming the specificity of inhibition of polio replication by GBF1 knock-down. We also performed the same experiment in the presence of a broad range caspase inhibitor zVAD-fmk, to prevent possible depletion of cells transfected with polio replicon due to apoptosis, known to be triggered when polio replication is suppressed [34]. Treatment of cells with zVAD-fmk did not change the reduction of polio replication in cells with knocked down GBF1 expression (not shown). Together, these results show that polio RNA replication strongly depends on the activity of GBF1.

### Expression of constitutively activated Arf1 Q71L mutant does not rescue polio replication from GBF1 loss of function

Since Arf activation by GBF1 appeared to be essential for polio replication, we tested whether expression of Arf1 Q71L, a mutant Arf1 protein that manifests increased affinity for GTP and therefore does not require GEF-mediated nucleotide exchange to be activated [35], can rescue polio replication under conditions of suppressed GBF1 activity. Figure 3C shows that expression of this Arf mutant did not support polio replicon replication in the presence of BFA, nor did expression of this mutant restore polio replication in cells treated with anti-GBF1 siRNA (not shown). These data are consistent with previous analyses of the Arf Q71L protein. Expression of this mutant prevented BFA-induced Golgi breakdown and loss of COPI from the membranes; however, because this Arf is unable to cycle, COPI became irreversibly locked on membranes and was not functional [35],[36],[37],[38]. Thus, viral RNA replication depends on the precise temporal and spatial regulation of GBF1-dependent Arf activation and cycling that is characteristic of this group of G proteins. The possibility that there is an Arf-independent GBF1 function that is required for virus replication also cannot be excluded.

### Expression of polio 3A protein stimulates GBF1-dependent Arf activation *in vitro*

**Figure 4:**
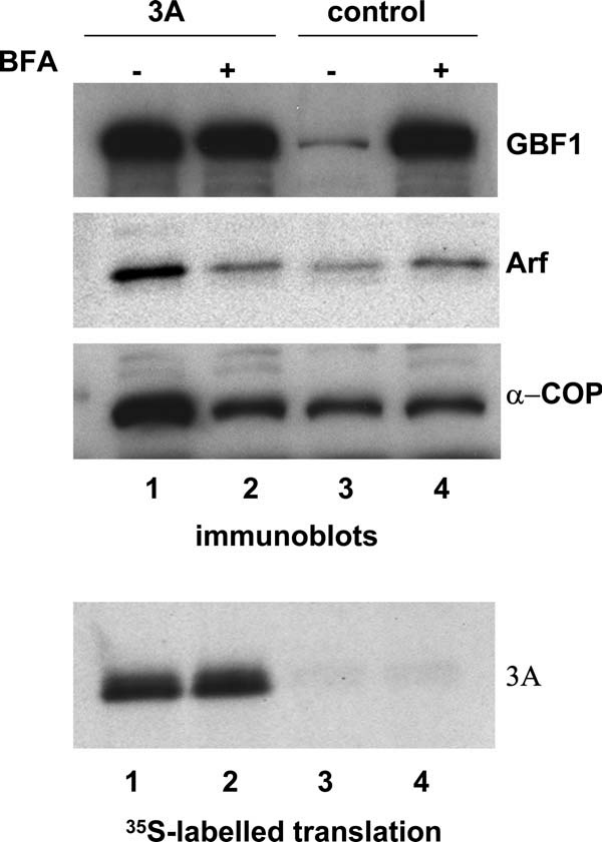
Synthesis of poliovirus protein 3A stimulates GBF1-dependent Arf activation in vitro. RNA coding for poliovirus 3A was translated in HeLa cell S10 extracts. The samples contained 80 µg/ml BFA where indicated (or the corresponding amount of solvent DMSO in other samples). The membranes were collected by centrifugation and assessed by western blot with anti-GBF1, anti-Arf and anti-COPI antibodies. Translation efficiency was monitored by labeling an aliquot of the translation reaction with ^35^S methionine (lower panel).

To confirm that 3A-induced recruitment of GBF1 to membranes results in Arf activation, we utilized an *in vitro* system that has been extensively exploited to reveal the biochemical machinery of poliovirus RNA replication [39],[40],[41],[42],[43]. RNA coding for poliovirus 3A was translated in HeLa cell extracts, and membranes were collected by centrifugation and analyzed by immunoblot to asses the proteins that were membrane-associated. As we showed previously [31] synthesis of 3A resulted in increased association of GBF1 with membranes (Fig. 4, GBF1 row; compare lanes 1 and 3). Interestingly, a significant accumulation of GBF1 on membranes was also observed in samples treated with BFA, independent of the synthesis of polio 3A protein (Fig. 4, GBF1 row; compare lanes 1, 2 and 4). However, only 3A synthesis resulted in increased amounts of Arf on the membranes. As expected, this Arf was activated, as evidenced by the recruitment of components of the COPI coatomer complex, a downstream effector of Arf activated through the GBF1-dependent pathway [44]. No Arf or COPI accumulation was observed in samples treated with BFA, regardless of the GBF1 association with membranes (Fig. 4 compare lanes 1, 2 and 4). These results clearly distinguish between the functional and abortive recruitment of GBF1 to membranes induced by 3A vs. BFA. Recruitment of GBF1 to membranes induced by poliovirus protein 3A leads to a productive cascade of Arf activation and COPI coatomer recruitment, consistent with our previous report [31] that increased levels of Arf-GTP steadily accumulate on membranes with time during poliovirus infection of HeLa cells.

### Interaction between poliovirus protein 3A and GBF1 determines sensitivity of virus replication to BFA

**Figure 5:**
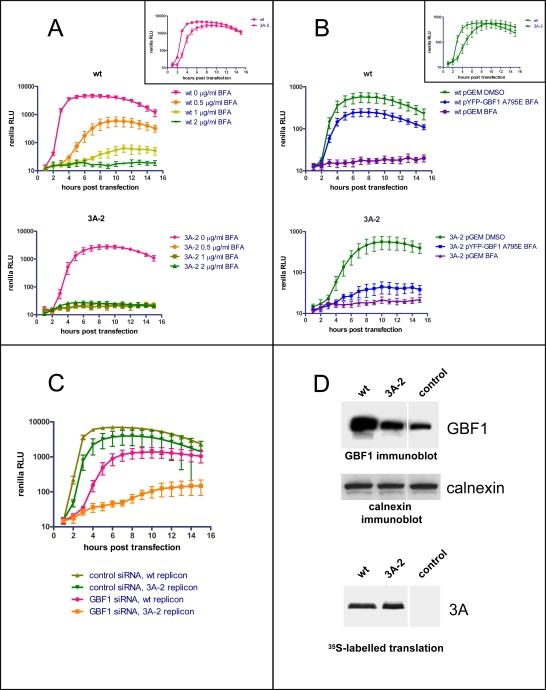
Mutation in poliovirus 3A protein increases viral sensitivity to BFA and decreases recruitment of GBF1 to membranes. A. HeLa cells were transfected with either wild type or 3A-2 mutant polio *Renilla* replicons and incubated in the presence of the indicated amounts of BFA. Luciferase was assayed as a measure of polio RNA replication. The inset shows a direct comparison of the wild type and 3A-2 replicons in the absence of BFA. B. HeLa cells were transfected with a plasmid expressing YFP-GBF1 A795E, a BFA-resistant mutant of GBF1, or a control vector. The next day the cells were transfected with either wild type or 3A-2 mutant polio Renilla replicon RNA and incubated with or without 1 µg/ml of BFA while RNA replication was monitored. The inset shows a direct comparison of the wild type and 3A-2 replicons in the cells transfected with an empty vector in the absence of BFA. C. HeLa cells were treated with either GBF1 or control non-specific siRNA for 2 days prior to transfection with the polio Renilla wild type or 3A-2 mutant replicons. Luciferase was assayed as a measure of polio RNA replication. D. RNA coding for either wild type 3A or 3A-2 mutant was translated in HeLa cell S10 extracts. The membranes were collected by centrifugation and assessed by western blot with anti-GBF1 antibodies. The same membrane was stripped and probed again with anti-calnexin antibodies as a loading control. Translation efficiency was monitored by labeling an aliquot of the translation reaction with ^35^S methionine (lower panel).

The 3A protein of coxsackievirus B3 (CVB3) was shown previously to interact with the N-terminus of GBF1 [21]. CVB3 and poliovirus are closely related enteroviruses, so we speculated that a similar interaction between poliovirus 3A protein and GBF1 might account for the observed requirement for GBF1 in poliovirus replication. First we examined the BFA sensitivity of a wild type poliovirus replicon and a mutant replicon containing an insertion of a Ser residue at position 15 in the 3A sequence. This mutant, called 3A-2, was shown previously to be defective in inhibiting the cellular secretory pathway [22],[45]. Wessels et al. showed that when the mutation corresponding to polio 3A-2 was introduced into CVB3, the resulting 3A protein manifested a severely decreased binding to GBF1 [46]. Replication of the 3A-2 replicon showed an approximately one-hour delay compared with wild type before the rapid phase of viral RNA synthesis begins. Although the final replication level was only slightly less than wild type in the absence of inhibitor (see insets, Fig. 5A and B), replication of the mutant was completely abrogated even at the lowest concentration of the inhibitor tested. The wild type replicon displayed intermediate levels of sensitivity at low BFA concentrations (Fig. 5A).

We also tested the level of rescue of replication of the 3A-2 mutant replicon in the presence of BFA by ectopic expression of GBF1. In this experiment we used our BFA-resistant A795E GBF1, to obtain the maximum level of rescue. As shown in Fig. 5B, the response of the 3A-2 mutant was significantly lower than that of the wild type replicon. Finally, we showed that GBF1 knockdown had a significantly greater inhibitory effect on the replication of the 3A-2 polio replicon than on the wild-type replicon. In this experiment we treated cells with GBF1 siRNA for a shorter period – 2 days instead of 3- to minimize cytotoxicity of the siRNA and therefore to be able to detect weaker possible replication of the 3A-2 replicon. Under these conditions the reduction in replication of the wild type replicon was clearly visible but, as expected, was less than 90% observed in the cells treated with GBF1 siRNA for 3 days, while replication of the 3A-2 replicon was inhibited to a much greater extent (Fig. 5C).

We have shown previously that synthesis of polio 3A protein in HeLa cell extracts results in accumulation of GBF1 on membranes ([31] and Fig. 4). To see if the pattern of BFA inhibition of replication of polio variants correlated with the ability of the corresponding 3A proteins to engage GBF1 on the membranes, we translated RNAs coding for wild type 3A and 3A-2 mutant in HeLa cell extracts, collected membranes with their associated proteins by centrifugation and assessed the amount of GBF1 by Western blot. Figure 5D shows that the amount of GBF1 bound to membranes after translation of 3A-2 mutant RNA was significantly less than after translation of wild type 3A RNA, correlating with the strong sensitivity of the 3A-2 replicon to BFA. Although the amount of GBF1 found associated with membranes upon translation of 3A-2 coding RNA varied in different batches of HeLa cell extracts, usually synthesis of 3A-2 mutant protein did induce some association of GBF1 with membranes compared with the background levels. Taken together, the data show that the 3A-2 mutant is more sensitive to BFA, more sensitive to depletion of GBF1 by siRNA kockdown, and is harder to rescue from BFA inhibition by providing ectopic GBF1. These properties are all consistent with low-affinity binding between 3A-2 and its target GBF1, whose activity is required for virus growth.

This analysis does not discriminate between a direct interaction of 3A with GBF1 and indirect activation of GBF1 translocation to membranes by a pathway triggered by 3A. To compare the strength of direct interaction between the two proteins we performed yeast two-hybrid studies with the soluble interacting domains of the wild type and 3A-2 mutant 3A polypeptides (amino acid residues 1–60) and GBF1. Our results (not shown) confirmed previous studies performed in a mammalian two-hybrid system, showing a strong interaction between wild type poliovirus 3A and GBF1 and the severe inhibition of such interaction for the 3A-2 mutation in the corresponding Coxsackie virus protein [20],[46]. Thus our data show that the sensitivity of poliovirus replication to inhibition by BFA correlates inversely with the strength of interaction of viral protein 3A and GBF1.

**Figure 6:**
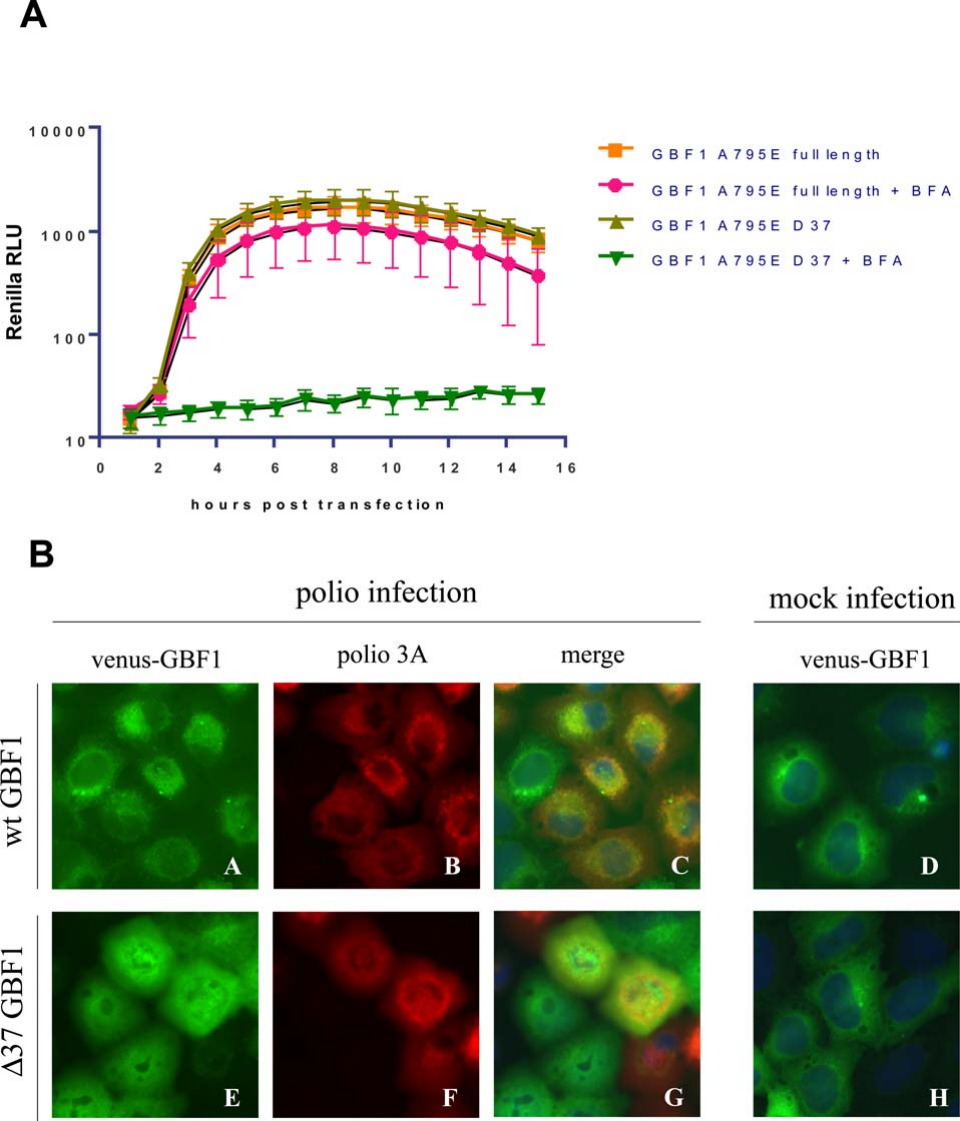
The N-terminal region of GBF1 is important for rescue of polio replication from BFA inhibition and for localization to sites of viral RNA synthesis. A. HeLa cells were transfected with vectors expressing YFP-GBF1 fusions with BFA-resistance mutation A795E with either the wild type N-terminus or with a deletion of 37 N-terminal amino-acids (Δ37). The next day the cells were transfected with polio *Renilla* replicon RNA and incubated in the presence or absence of 1 µg/ml BFA while monitoring viral RNA synthesis. B. HeLa cells were transfected with plasmids expressing Venus-GBF1 fusions with either wild type N-terminus (panels A–D) or with a deletion of 37 N-terminal amino-acids (Δ37) (panels E–H). The next day cells were infected with poliovirus at a multiplicity of 10 PFU/cells (panels A–C and E–G) or mock-infected (panels D and H), incubated for 4 h, fixed and processed for immunofluorescent staining with anti-polio 3A antibodies (red). Nuclear chromatin is stained with Hoechst 33342.

The domain attributed with binding to viral protein 3A was shown to reside in the N-terminal region of GBF1. A deletion of 37 N-terminal amino acids of GBF1 resulted in the loss of interaction with 3A protein from CVB3, as detected by co-immunoprecipitation experiments [46]. We tested this Δ37 mutation in GBF1 for its ability to rescue polio replication in the presence of BFA. To ensure as great a potential rescue level as possible, we combined the Δ37 mutation with the BFA-resistance mutation A795E, identified in GBF1 from BER-40 cells. Although expression of the YFP fusions of the full length A795E GBF1 was very efficient in rescuing replication of polio replicon RNA in the presence of the inhibitor, the truncated variant of GBF1 was completely ineffective (Fig. 6A). We also tested whether this truncated variant of GBF1 combined with the A795E mutation could confer BFA resistance to HeLa cells. The survival experiments did not show any protection of cells expressing this GBF1 variant from BFA as opposed to cells expressing full length A795E GBF1 (not shown). Therefore the N-terminal region of GBF1 provides some important function(s) required for BFA resistance for both virus replication and cell survival.

We monitored the distribution of Venus fusions of wild type GBF1 and Δ37 mutant GBF1 in cells infected with poliovirus. The localization of both proteins in mock-infected cells was virtually identical: they were associated with cytoplasmic ER-like structures with some slight concentration in the perinuclear area (Fig. 6B, panels D and H). In infected cells the two proteins behaved very differently. As we reported previously, wild type GBF1 relocalized to sites of poliovirus replication, visualized by staining of 3A protein (Fig. 6B panels A–C) [31]; the Δ37 mutant GBF1 showed no apparent association with membrane structures, and displayed the diffuse fluorescence typical of a soluble protein (Fig. 6B panels E–G). A characteristic feature of infected cells expressing the truncated GBF1 mutant was the positive staining of the nucleus, which was always spared in cells expressing wild type GBF1 (Fig. 6B panels A, E) and in mock-infected cells expressing either protein (Fig. 6B panels D, H). The difference between localization of wt GBF1 and the Δ37 mutant in infected cells is likely due to the inability of the deletion mutant to be tethered to membranes because of loss of a domain responsible for interaction with protein binding partners, including polio protein 3A.

### BFA inhibits function of poliovirus replication complexes but not virus-induced remodeling of cellular membranes

**Figure 7:**
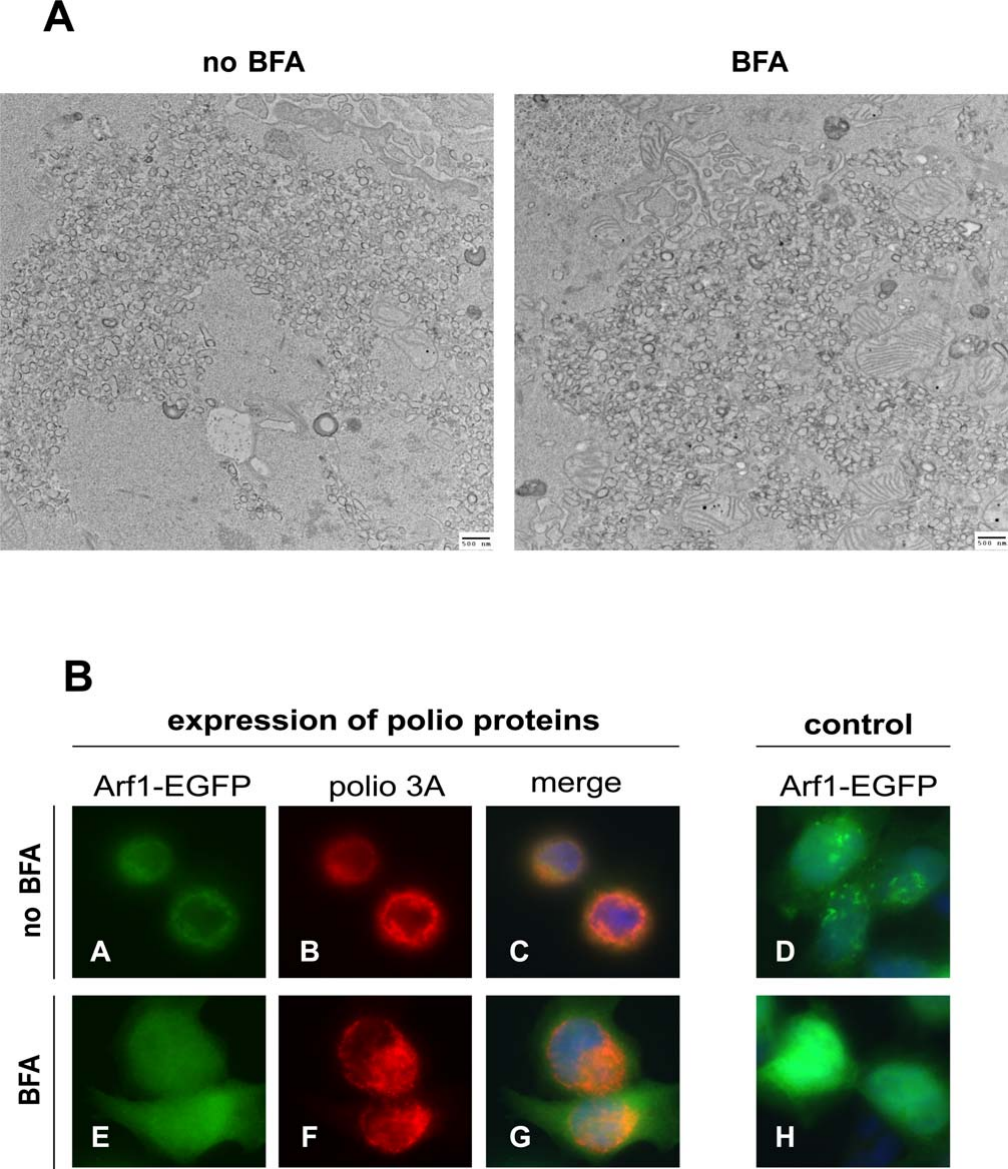
Polio-induced vesicles form in the presence of BFA, but fail to induce Arf1 relocalization. A. Polio non-structural proteins were expressed from non-replicating RNA. The cells were incubated in the presence or absence of 2 µg/ml of BFA. An equivalent amount of DMSO solvent was added to the incubation medium in the sample without BFA. After 4.5 h incubation, cells were fixed and processed for electron microscopy. B. Poliovirus non-structural proteins were expressed from non-replicating RNA in HeLa cells expressing Arf1-EGFP fusion. The cells were incubated in the presence of 2 µg/ml of BFA where indicated (panels E–H). An equivalent amount of DMSO solvent was added to the incubation medium in samples without BFA (panels A–D). To observe the localization of Arf1-EGFP fusion protein in cells without expression of polio proteins, an empty plasmid was substituted for the polio cDNA-bearing plasmid (panels D and H).

GBF1-induced activation of Arf is required for formation of COPI-coated vesicles in the traffic between ER and cis-Golgi of the cellular secretory pathway [44]. The association of activated Arf with membranes also recruits numerous effector proteins and changes membrane properties due to activation of lipid-modifying enzymes such as phospholipase D [15],[47]. Although polio RNA replication clearly requires active GBF1, the Arf-GTP generated by this GEF may participate in two not necessarily mutually exclusive processes: the remodeling of cellular membranes into characteristic polio-induced vesicles, and/or the generation of a favorable membrane microenvironment for RNA replication. Since virus replication is inhibited by drugs that prevent Arf activation, such as BFA, we attempted to determine whether BFA affected either of these two potential inputs of Arf into poliovirus replication. To this end, we utilized a non-replicating poliovirus RNA where the cognate regulatory sequences in the 5′ non-translated region were substituted with an IRES from encephalomyocarditis virus (EMCV) to express polioviral proteins. This non-replicating RNA was expressed from a plasmid in HeLa cells by T7 RNA polymerase produced from a recombinant vaccinia virus. In this case accumulation of poliovirus-specific RNA is not dependent on replication, and the synthesis of polio proteins is not affected by the presence of brefeldin A. This construct was used previously to show that synthesis of poliovirus proteins, not RNA replication, is sufficient to form the vesicular structures morphologically indistinguishable from those found in poliovirus-infected cells [48]. When the transfected cells were examined by electron microscopy, specific polio-induced vesicles were observed to have formed, both in the presence and the absence of BFA, thus arguing that Arf activation is not necessary for the morphological development of these structures (Fig. 7A). To monitor the distribution of Arf in cells expressing poliovirus proteins in the presence of BFA we performed a similar experiment in cells expressing an Arf1-EGFP fusion protein. As seen in Fig. 7B, staining of poliovirus protein 3A revealed that regardless of the presence of BFA, the viral protein was localized in the characteristic perinuclear ring of vesicle-like structures (compare panels B and F), while Arf translocated to this region only in the absence of the inhibitor (compare A and E; C and G). As expected, Arf translocation to the virus-induced vesicular structures did occur in the presence of BFA in cells transfected to express the BFA-resistant A795E GBF1 mutant (not shown).

**Figure 8:**
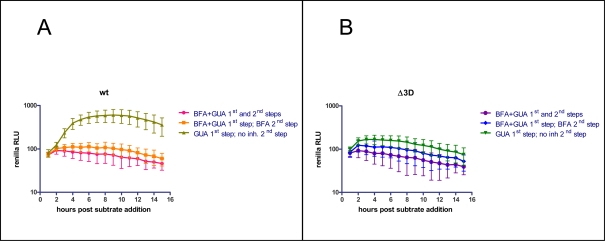
Vesicles formed in the presence of BFA do not support viral RNA replication. A. Polio *Renilla* replicon RNA was synthesized in cells in the presence of 2 mM guanidine-HCl and 2 µg/ml BFA as indicated. These conditions allowed for RNA translation and formation of vesicles, but prevented RNA synthesis. After 4.5 h the medium was changed to remove guanidine-HCl and allow RNA synthesis, and *Renilla* luciferase activity was monitored as a measure of viral RNA replication. BFA and guanidine were present at both steps in the control sample. B. The same experiment was performed with a replicon containing a deletion in the polymerase gene.

The results of this experiment show that in the presence of BFA, when GBF1-dependent Arf activation could not occur, it is possible to form polio-induced membranous structures morphologically indistinguishable from those developed in the absence of the inhibitor. To determine whether these structures were capable of supporting polio RNA replication, we allowed them to form in the presence of absence of BFA with a replication-competent RNA, and then measured their subsequent ability to synthesize RNA. Viral proteins were synthesized from polio RNAs generated by T7 RNA polymerase supplied by recombinant vaccinia virus. To ensure that equal amounts of proteins were produced during the stage of membrane remodeling with and without BFA, both samples were incubated in the presence of 2 mM guanidine-HCl, a specific and reversible inhibitor of polio RNA replication that prevented amplification of viral RNA template. The cells were incubated for 4.5 hours, the time that we found in the previously-described electron microscopy studies to be sufficient for vesicular structures to form. The presence of guanidine-HCl during this time blocked RNA replication from starting even if competent replication complexes had formed. After 4.5 hours, the guanidine-HCl was removed to allow viral RNA synthesis to proceed from the pre-formed protein-membrane complexes. Figure 8A shows that polio RNA replication was detected only when the initial incubation was performed without BFA, showing that the vesicular structures associated with viral replication proteins formed when Arf activation was inhibited were unable to support viral RNA synthesis despite their similar morphological appearance. To confirm that the observed increase in *Renilla* signal from the cells whose replication complexes were formed in the absence of BFA was not simply due to healthier cells incubated without BFA, we performed a similar experiment with a plasmid coding for a replication-defective polio RNA containing a deletion in the polymerase gene. In this case no differences were seen between the samples regardless of their BFA treatment (Fig. 8B). These data demonstrate that the BFA-sensitive (and Arf activation-dependent) step in polio replication is not the remodeling of host membrane structures, but the ability of those structures to function in viral RNA replication.

## Discussion

Viruses ultimately must depend on cellular structures and factors for their replication. Often host proteins in infected cells are diverted to perform their normal function(s) in a new microenvironment or with modified specificity. In this paper we show that replication of poliovirus strongly depends on the activity of a cellular protein, GBF1. GBF1 activates the small cellular GTPase, Arf1, by exchanging Arf-bound GDP for GTP to regenerate the active form of Arf. In uninfected cells, GBF1 is required for specific steps during the transfer of proteins and membranes through the secretory pathway, from ER through the Golgi to plasma membrane or endosomes. Activation of Arf by GBF1 occurs during formation of COPI-coated vesicles from the ER-Golgi intermediate compartment (ERGIC). This activity of GBF1 is inhibited by BFA which binds and stabilizes the Arf1-GDP-GBF1 intermediate complex and thus prevents GBF1 from performing multiple rounds of Arf activation [28],[29]. Replication of poliovirus is also sensitive to this inhibitor [10],[11]. We demonstrate that GBF1 can rescue poliovirus infection from inhibition by BFA, and that reduced interaction between GBF1 and viral protein 3A increases the sensitivity of poliovirus infection to BFA. Inhibition of polio replication by BFA is specific for the particular host cell, and this specificity is determined by the sequence of the catalytic Sec7 domain of GBF1 in the cell. We identified a new mutation in GBF1, A795E, that renders the factor resistant to BFA. Expression of this BFA-resistant GBF1 conferred BFA resistance to HeLa cells and allowed efficient rescue of poliovirus replication in the presence of BFA. This mutation generates a single amino acid substitution at a residue that is very close to the BFA binding site identified on the crystal structure of the complex between the GEF Sec7 domain, BFA and Arf-GDP [49],[50]. The crystal structure of a modified Arno Sec7 domain complexed with Arf1 and BFA shows that the corresponding residue (A157) participates in direct van der Waals interaction with BFA. Interestingly, another amino acid in the Sec7 domain of GBF1, M832, whose substitution to L also makes GBF1 resistant to BFA, is located very close to A795 in the crystal structure and also participates in van der Waals contact with BFA [29],[49].

Previous work from this laboratory demonstrated that two other high molecular weight GEFs that activate Arf, BIG1/BIG2, are recruited to polio replication complex membranes, both in vitro and in infected cells [31]. Recruitment of BIG1/BIG2 was mediated by viral protein 3CD, independent of 3A's recruitment of GBF1. Since the activities of these two GEFs are also sensitive to BFA, the data described in this report suggest that BIG1/BIG2 also are not involved in the morphological development of replication vesicles. It is not yet clear whether or what role these GEFs play in this complex process [17], since any or all of them might perform BFA-insensitive functions that could affect membrane remodeling or other aspects of polio replication.

Wessels et al. have performed elegant studies on the fate and consequences of expressing just the 3A protein from polio or Coxsackie virus B3 in mammalian cells. They found loss of COPI coatomer complex on membranes and reduction of activated Arf [21]. They also showed that 3A interacts directly with GBF1, and concluded that this interaction resulted in inhibition of the Arf-activating property of GBF1 when 3A was expressed by itself in mammalian cells. The single amino acid insertion in the 3A-2 mutant caused the viral protein to lose detectable binding to GBF1 and therefore did not inhibit GBF1's GEF activity [20],[46]. It was proposed that this 3A-induced inhibition of GBF1 GEF activity was responsible for shutting down the secretory pathway in infected cells. In the course of virus infection, 3A protein is synthesized together with other viral proteins and is likely involved in interactions with other viral products that may significantly modify the outcome of its interactions with cellular proteins [51],[52]. Our previous data showed that the amount of Arf-GTP steadily increased during the course of infection [31]; thus at least overall GEF activity in infected cells is not inhibited. Moreover Gazina et al. demonstrated that components of COPI coats, whose association with membranes is directly dependent on GBF1-induced Arf activation, are associated with replication complexes of echovirus 11, a related picornavirus that is sensitive to BFA [53]. A genetic screen of Drosophila cells identified the COPI coatomer as a host factor essential for growth of another picornavirus-like virus, Drosophila C virus [54]. These data do not support the notion that GBF1 activity is inhibited in infected cells. Our data presented here directly show that GBF1 is necessary for poliovirus replication and that only expression of catalytically active GBF1 can rescue poliovirus replication in the presence of BFA. Moreover we showed that synthesis of 3A results in stimulation of GBF1-dependent activation of Arf *in vitro*. These latter experiments are the most difficult to reconcile with the results from the van Kuppeveld laboratory [21] since they demonstrate stimulation rather than inhibition of Arf activation even in the absence of other viral proteins. Collaborative studies in both laboratories are currently in progress to attempt to understand these apparently conflicting results. Our data also suggest that although the 3A-2 mutant may manifest a much weaker interaction with GBF1, it still retains some residual ability to induce association of this protein with membranes. We propose that interaction of 3A with GBF1 diverts its activity from its normal function in the secretory pathway to sites of polio replication where it functions in poliovirus (and likely other BFA-sensitive picornaviruses) replication. This diversion to viral replication complexes would likely result in inhibition of cellular secretion. Inhibition of cellular secretion leads to reduced presentation of antigens on the surface of infected cells as well as reduced release of cytokines [22],[55]. Thus, effective subversion of a cellular pathway may provide a double benefit for the virus by sustaining genome replication in the cell as well as inhibiting a pathway important for the organism's defense against infection.

Inhibition of the cellular secretory pathway was suggested to provide an advantage for replication of the virus in an animal host, but was believed to be dispensable for replication in cells culture. Although the 3A-2 mutation was found to strongly reduce poliovirus's ability to inhibit cellular secretion [23], it was reported not to interfere significantly with propagation of the virus in cell culture. On the other hand, when the corresponding mutation was introduced into CVB3, the mutant was less pathogenic in mice [21]. The mechanism of the apparent attenuation remains to be elucidated.

What roles do GBF1-dependent reactions play in poliovirus replication? GBF1 normally participates in the formation of COPI-coated vesicles on ERGIC structures, and poliovirus replication complexes form on membranous structures that resemble clusters of heterogeneously sized vesicles. Thus, GBF1 may possibly be involved in remodeling host membranes into those structures. In poliovirus-infected cells, however, development of infection very rapidly results in complete reorganization of cellular organelles into specific membranous vesicles, so that ER, ERGIC and other structures are no longer detectable [2], although some early secretory pathway steps could still be observed in infected cells [56]. Therefore infected cells very rapidly lose the normal morphological substrate for formation of COPI-like vesicles. Supporting this idea is our observation that the Δ37 mutant of GBF1 is distributed like a soluble protein in infected cells while it is indistinguishable from the wt GBF1 in mock-infected cells. Apparently GBF1 in polio-infected cells is no longer retained on membranes through its normal interactions, such as binding to Rab1b and/or p115 [57],[58], but wt protein is tethered on remodeled membrane structures through binding to polio protein 3A, while the Δ37 GBF1 mutant is unable to interact with 3A and therefore behaves like a soluble protein. Poliovirus infection is known to induce rapid degradation of nuclear pores and consequent leakiness of the nuclear envelope that allow even high molecular weight soluble proteins to freely penetrate the nuclear envelope in both directions [59],[60]. Another set of data also suggests that the BFA-sensitive (and therefore the GBF1-dependent) step in poliovirus replication is not remodeling of membranes but rather proper functioning of the replication complexes. Poliovirus infection is sensitive to addition of BFA at every stage of the replication cycle ([11]; our unpublished observations), which would be difficult to reconcile with the requirement of such activity only for the morphogenesis of replication structures. Our experiments presented here directly show that BFA does not preclude formation of characteristic vesicle-like structures. The similar appearance by low-power electron microscopy of the membrane structures formed by viral proteins in the presence and absence of BFA was surprising; however, the apparent similarity does not imply that these structures are biochemically and functionally similar, as evidenced by the differences in Arf1 localization and very likely numerous other markers including the effectors normally recruited by Arf. Those structures are unable to support polio RNA replication when the inhibitor is removed. Association of activated Arf with membranes is known to induce binding of many effector proteins and coat complexes and to activate membrane-modifying enzymes [15],[47]. It is likely that in the polio-induced vesicles, Arf's role could be to bring to the membranes other host proteins that participate in replication of the viral genome or in regulating the lipid composition of these structures to make them suitable for assembly of functional replication complexes. Interestingly, expression of the Arf1Q71L mutant that has increased affinity for GTP and therefore is always in an “activated state” was not able to restore polio replication from either inhibition of GBF1 activity by BFA or from knock-down of GBF1 expression by siRNA. This result may indicate that GBF1 performs some other function in polio replication, unrelated to Arf activation, but the most likely explanation of this inability of the Arf1 Q71L mutant to rescue polio replication is that the replication process requires Arf that can perform normal cycling between its GTP- and GDP-bound form, while Arf1 Q71L is constantly activated and bound to membranes [35],[36],[37],[38].

All positive strand RNA viruses remodel host membranes into novel structures where replication complexes are assembled, and RNA replication of at least some of them was shown to be sensitive to BFA [53],[61]. It is likely that BFA-sensitive Arf activation is the cellular pathway exploited by diverse groups of RNA viruses.

## Materials and Methods

### Cells

HeLa cells were maintained in Dulbecco's Minimum Essential Medium, high glucose modification, supplemented with 1 mM sodium pyruvate and 10% heat-inactivated fetal bovine serum. BFA-resistant BER-40 cells and their parental Vero cell line were kindly provided by T. Oda, University of Nagasaki, Japan. They were grown in Eagle's Minimum Essential Medium supplemented with 10% heat-inactivated fetal bovine serum.

### Plasmids

Plasmids pXpA-3A and pXpA-3A-2, coding for poliovirus wild type 3A and 3A-2 mutant, respectively, have been described [19]. The pXpA-RenR plasmid, encoding a poliovirus replicon with the *Renilla* luciferase gene substituting for the capsid coding sequence was previously described [31]. Plasmid pXpA-RenR 3A-2 was obtained by point mutagenesis, and the mutagenized fragment was verified by sequencing. Plasmid pTM-PV-2A-3′, used for expression of poliovirus non-structural proteins under translational control of the EMCV IRES was generously provided by N. Teterina in our laboratory. Plasmid pXpA-RenR Δ3D is a derivative of pXpA-RenR with a deletion of 190 nt in the polio polymerase sequence. Plasmid pYFP-GBF1, pYFP-GBF1 E794K and pVenus-GBF1 for expression of GBF1 derivatives have been previously described [29]; pVenus-GBF1 Δ37, derived from pVenus-GBF1, was constructed by T. Niu. Plasmid pArf1-EGFP for expression of Arf1-EGFP fusion was described elsewhere [36]. Plasmid pArf1Q71L-CFP was a gift from N. Altan-Bonnet (Rutgers University, New Jersey). Plasmid pCMV-Gluc used for the secretion rescue experiment was purchased from New England Biolabs.

### Antibodies

Rabbit polyclonal anti-GBF1 antibodies were a gift from N. Altan-Bonnet, Rutgers University, New Jersey. Anti-BIG2 rabbit antibodies were generously provided by M. Vaughan, NHLBI, NIH. Anti-polio 3A mouse monoclonal antibody was a gift from K. Bienz, University of Basel, Switzerland. Rabbit polyclonal anti-COP α and γ were a gift from F. van Kuppeveld, Radbout University, the Netherlands. Mouse monoclonal anti-Arf antibodies recognizing all species of mammalian Arf except Arf4 were from Affinity Bioreagents. Mouse monoclonal anti-actin antibodies conjugated with horse radish peroxidase were from Sigma. Mouse monoclonal anti-GFP antibodies were form Clontech. Secondary antibody Alexa Fluor 594 conjugates used in immunofluorescence were from Molecular Probes. Secondary antibody horse radish peroxidase conjugates used in Western blots were from Amersham.

### Poliovirus propagation assay

Vero or BER40 cells grown on 6 cm plates were infected with poliovirus at multiplicity of 10 PFU/cell and incubated in the presence of 2 µg/ml of BFA for 6 hours. The cells were subjected to three freeze-thaw cycles to release intracellular virus, and virus yield was determined by standard plaque assay on HeLa cell monolayers.

### Sequencing of Sec7 domains of BIG1, BIG2 and GBF1

Poly(A)-containing RNA from Vero and BER-40 cells was isolated with Oligotex mRNA Mini Kit (Qiagen) according to the manual. Reverse transcriptase reaction with oligo-dT primer was performed with MonsterScript 1^st^-Strand cDNA Synthesis kit (Epicentre Biotechnologies). Sec7 –containing fragments were amplified by PCR using Phusion High Fidelity PCR kit (Finnzymes). Big1 Sec7 PCR primers: GATCGGTCGACACTAGTAAATGATCTATC (forward) GATCGAAGCTTCTTAAGAAATCCTTCTGG (reverse); Big2 Sec7 PCR primers: AGTCAGCATGCATTTAAATGCTGCTAAC (forward), GACTGAAGCTTACCGGTTCCTATCAG (reverse); GBF1 Sec7 PCR primers: TCAGAAAGCTTATGGAGATCATCACTGTGG (forward) CAGAGAATTCCTTAAGCAGAGACTTAGTGTC (reverse). Sequencing primers are available upon request.

### Polio replicon assays

Polio replicon RNA was transfected into HeLa cells grown in 96 well white plates with a clear flat bottom (Costar) at 10 ng/well with Trans-it mRNA transfection reagent (Mirus) according to the manufacturer's recommendations. Incubation media contained 60 µM live cell Renilla substrate Endu-Ren (Promega) and BFA where indicated. Control samples contained an equivalent amount of DMSO, used as solvent for BFA. Renilla light signal was recorded with SpectraMax M5 multi-well plate reader (Molecular Devices). Each point on a graph is an average of measurements obtained from at least 16 wells.

### siRNA

GBF1 siRNA CAACACACCUACUAUCUCU was obtained from Dharmacon. Silencer Negative control #1 siRNA was purchased from Ambion. HeLa cells were seeded at 10 000/well in a 96-well plate, transfected the next daywith Dharmafect1 transfection reagent (Dharmacon) according to the manufacturer's recommendations and incubated for 3 more days before the polio replication experiments.

### BFA toxicity assay

HeLa cells were plated at 20 000 per well in a 96-well plate and transfected with plasmids with Lipofectamine LTX (Invitrogen) according to the manufacturer's recommendations. The next day the medium was supplemented with 100 ng/ml of BFA and the cells were incubated for two more days with medium change approximately every 8 hours to ensure constant presence of the inhibitor. Cell viability was assessed with CellTiter-Glo luminescent cell viability assay kit (Promega).

### Secretion rescue assay

HeLa cells in a 96 well plate were transfected with pGEM-3Z (control), pYFP-GBF1 wt, or pYFP-GBF1 A795E and pCMV-Gluc vector (4∶1 mass ratio). The next day the cells were washed with serum-free medium and incubated with BFA (1 µg/ml) or DMSO for 5 h in normal growth medium (75 µl/well). A portion (20 µl) of the medium from each well was assayed with 20 µl of Gaussia Luciferase assay solution (New England Biolabs).

### In vitro translation

HeLa S10 extracts for translation reactions were prepared as described [43] but were not treated with micrococcal nuclease. Translation reaction mixtures of 50 µl included 2.5 µg of RNA transcripts. An aliquot of 9 µl from each reaction mixture was mixed with 1 µl of Redivue VIral methionine (Amersham) and incubated for 3.5 h at 34°C, after which one-fourth of the material was resolved by sodium dodecyl sulfate-polyacrylamide gel electrophoresis (SDS-PAGE) mini-gel for visualization of translation products. The remaining 40 µl were also incubated for 3.5 h at 34°C and then centrifuged for 20 min at 16,000×*g* at 4°C. Pellets were assayed by Western blot with the ECL Advance system (Amersham) according to the manufacturer's recommendations.

### Vaccinia T7 expression system

#### For electron microscopy

HeLa cells were plated in a 12-well plate with Thermanox plastic coverslips (Nalge Nunc) at 200 000 cells/well, one day prior to infection with vaccinia virus VT7-3, expressing T7 RNA polymerase [62] at a multiplicity of 10 PFU/cell. After one hour the cells were transfected with plasmids with Lipofectamine LTX (Invitrogen) and incubated for 4.5 hours. Cells were then washed with PBS and placed in fixative (2.5% glutaraldehyde /4% paraformaldehyde in 0.1 M sodium cacodylate buffer) and stored at 4°C. Transmission electron microscopy was performed at Rocky Mountain Laboratory (NIAID NIH).

#### For immunofluorescent microscopy

HeLa cells were plated on coverslips in a 12-well plate at 200 000 cells/well, one day prior to Lipofectamine LTX transfection with a 4∶1 mass mixture of pArf1-EGFP and pTM-2A-3′, respectively. After 16 hours incubation, the cells were infected at 10 PFU/cell with vaccinia VT7-3 virus for 1 hour and than incubated for 4.5 hours and processed for immonofluorescent microscopy.

#### For replication assay

HeLa cells were plated in a 96-well plate at 20 000 cells/well. Vaccinia infection and DNA transfection were performed essentially the same way as for electron microscopy, but the cells were transfected with linearized plasmids pXpA-RenR or pXpA-RenRΔ3D. Where indicated, inhibitors were added simultaneously with plasmid transfection. The cells were incubated for 4.5 hours, the medium was changed to include 60 µM Endu-Ren live cell Renilla substrate (Promega) and inhibitors where indicated. Renilla light signal was recorded with a SpectraMax M5 multi-well plate reader (Molecular Devices).

### Immunofluorescent microscopy

Cells grown on glass coverslips were fixed with 4% paraformaldehyde-phosphate-buffered saline (PBS) for 20 min, washed with PBS 3 times and stored in PBS at 4°C. Before staining the cells were permeabilized with 0.2% Triton X-100 in PBS for 5 min and washed 3 times with PBS. The cells were then incubated in 3% nonfat dry milk solution for 1 h to block nonspecific binding sites. This solution also was used for dilution of primary and secondary antibodies in which cells were sequentially incubated for 1 h. 10 ng/ml of Hoechst 33342 was added to the first blocking solution to stain nuclear chromatin. Images were taken with Leica DMIRE microscope. Digital images were processed with Adobe Photoshop software.
